# Long non-coding RNAs in the CFTR locus: New transcriptional regulators of CFTR gene expression

**DOI:** 10.1016/j.omtn.2026.103039

**Published:** 2026-07-27

**Authors:** Jessica Varilh, Karine Deletang, Arnaud Bourdin, Prisca Boisguerin, Magali Taulan-Cadars

**Affiliations:** 1 PhyMedExp, University Montpellier, INSERM U1046, CNRS UMR 9214, 34093 Montpellier, France; 2 University Montpellier, 34090 Montpellier, France; 3 CHU Arnaud de Villeneuve, 34295 Montpellier, France

**Keywords:** MT: Non-coding RNAs, cystic fibrosis, gene expression regulation, lncRNA, microRNA, lncRNA-miRNA interaction network

## Abstract

Cystic fibrosis (CF) is caused by mutations in the tightly regulated cystic fibrosis transmembrane regulator (CFTR) gene. The CFTR gene is weakly expressed in adult tissues, and its locus contains elements that regulate its mRNA and protein expression. Here, we identified new long non-coding RNAs (lncRNAs) transcribed from the CFTR locus that are differentially expressed in human tissues. In particular, we focused on the CF006 lncRNA, which stimulates CFTR transcription and increases mRNA and protein expression and was strongly downregulated in bronchial epithelium reconstituted from CF-derived airway epithelial cells. Our findings also suggest that CF006 may act directly by binding to the CFTR promoter and/or indirectly by affecting the local binding of transcription factors. Moreover, CF006 lncRNA expression was negatively regulated by miR-145, a microRNA (miRNA) upregulated in CF. Altogether, our findings suggest that the lncRNA CF006 is a new *cis*-regulatory element of CFTR expression and, therefore, a candidate therapeutic target for CF.

## Graphical abstract

**Figure:**
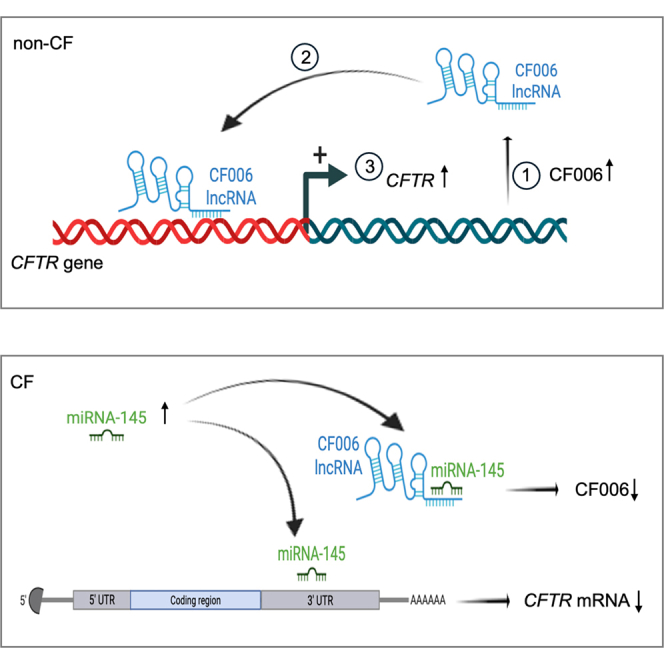

## Teaser

This study identifies new long non-coding RNAs within the CFTR locus and reveals that CF006 enhances CFTR expression by directly binding its promoter and interacting with transcription factors. CF006 is suppressed in cystic fibrosis and regulated by miR-145, highlighting a new regulatory axis and potential therapeutic target.

## Introduction

Cystic fibrosis (CF) is the most common hereditary genetic disease in White populations and is caused by mutations in the *cystic fibrosis transmembrane regulator* (*CFTR*) gene, which encodes a chloride channel expressed at the apical surface of epithelial cells.^1^^,^^2^^,^^3^ More than 2,000 alterations have been described in the *CFTR* gene, and the most common mutation, the deletion of the phenylalanine residue at position 508 (p.Phe508del), has been identified in about 67% of CF alleles. CF modulators, which target CFTR protein defects, are now approved for 85%–90% of patients with CF, but CF management remains challenging. Identifying elements that regulate *CFTR* expression is a way to improve the efficiency of currently available CF modulators and possibly broaden the number of treatable mutations or increase the efficacy of emerging gene therapies.

Indeed, the mechanisms governing the temporal and spatial expression of *CFTR* remain poorly understood.^4^^,^^5^ Its expression is restricted to epithelial cells of airways, pancreas, small intestine, and male genital ducts.^6^ In the lungs, *CFTR* transcripts are strongly detected in the fetus from week 12 to 24 of pregnancy; then, its expression progressively decreases and remains low during adult life.^7^ Much research has focused on identifying the elements that finely and tightly regulate *CFTR* expression. Its promoter region lacks a TATA box, is quite GC rich, and harbors AP-1, C/EBP, and FOXA binding motifs, as well as several transcriptional start sites (TSSs).^8^^,^^9^^,^^10^^,^^11^ The predominant TSS drives the majority of *CFTR* transcripts,^3^ but alternative TSSs can be used in cell lines with low *CFTR* levels. *Cis*-acting elements 632 bp upstream of the TSS and *CFTR* transcripts from even more distant TSSs, between −868 and −794, have been described in pancreatic and colon cell lines.^12^^,^^13^ Within the minimal promoter, several transcription factors (TFs) regulate *CFTR* expression and participate in its tissue-specific expression.^7^^,^^8^^,^^14^^,^^15^^,^^16^ Moreover, some key TFs implicated in airway development contribute to maintaining the low *CFTR* expression levels after birth.^7^ Other motifs in the 3′ UTR of *CFTR* mRNA that are targeted by small non-coding RNAs are also involved in the fine-tuning of *CFTR* expression.^7^^,^^17^^,^^18^^,^^19^^,^^20^ This class of small RNAs acts in synchrony with TFs to maintain the weak *CFTR* level in mature cells. In addition, studies of 3D chromosome organization have shown that the *CFTR* locus is included in a topologically associated domain (TAD) that also includes neighboring genes. The TAD boundaries include *cis*-regulatory elements and insulators, such as CTCF. Moreover, the *CFTR* promoter displays intra-TAD looping only in cells that express *CFTR*.^14^^,^^20^^,^^21^^,^^22^^,^^23^ This architecture seems to be essential for the correct expression of *CFTR* and its neighboring genes and for preventing contacts with neighboring regions. Previous studies have suggested that besides CTCF binding, additional mechanisms are implicated in defining topological domains and proposed that enhancer long non-coding RNAs (lncRNAs) block gene looping.^24^^,^^25^

In the past decade, many studies have unveiled the relevance of lncRNAs in gene regulation and diseases, a new class of non-coding RNAs initially considered transcriptional noise.^26^^,^^27^^,^^28^ These transcripts harbor mRNA features, such as 5′ cap and alternative splicing and a size >200 nt but modest conservation across species depending on their activities.^28^ lncRNAs are more abundant than protein-coding transcripts and are classified according to their genomic location (overlapping, intronic, or promoter-associated lncRNAs) and their functional activity.^29^^,^^30^ Although most lncRNAs have not been functionally characterized, they display a wide range of roles in the nucleus and cytoplasm, including chromosomal organization, chromatin modifications, transcription, and post-transcriptional regulation.^26^^,^^27^ In particular, lncRNAs can recruit and interact with key transcription partners and play a role in transcriptional control via *cis*- or *trans*-mechanisms. For instance, *HOTAIR*, the first described lncRNA, acts as a repressor and a scaffold for protein complexes.^31^^,^^32^ Collectively, lncRNAs have been investigated as regulatory players in the control of genes showing tissue-specific expression. Their regulatory role is linked to their ability to interact with DNA, RNA, and proteins, and some lncRNAs have been reported to control the expression of nearby genes by affecting their transcription through their *cis*-activating role.^33^

In CF, a microarray-based genome-wide lncRNA expression profiling study showed the dysregulation of several lncRNAs in CF bronchial epithelium^34^ and in primary bronchial cells infected with *Pseudomonas aeruginosa*, which colonizes the lungs in adults with CF.^35^ More recently, the same approach allowed the identification of other lncRNAs that are dysregulated in the lung airway and parenchymal tissues from patients with CF compared with controls without CF.^36^ Another way to study lncRNAs is to focus on the *CFTR* extended locus. This allowed the identification of an lncRNA (named *BGas*) that is transcribed in the antisense orientation from intron 11 of *CFTR* and that negatively controls CFTR transcription.^37^^,^^38^

In the present study, we thoroughly investigated the extended *CFTR* locus (i.e., the 300-kb-long sub-TAD) to identify potential non-coding RNAs with enhancer or repressive functions in CF bronchial cells. Using *in silico* analyses and cell experiments, we identified an lncRNA, *CF006*, that increases *CFTR* gene expression. We also found that the *CF006* lncRNA directly binds to the *CFTR* promoter, where it may interact with some TFs that have binding sites in the same region of the *CFTR* promoter. Moreover, in the *CF006* sequence, we identified a binding motif for miR-145, a microRNA (miRNA) that is strongly upregulated in CF. These findings suggest that *CF006* is a new candidate involved in the sophisticated control of *CFTR* expression and offer new opportunities for improving therapeutic approaches.

## Results

### Identification in the CFTR locus of new lncRNAs with tissue-specific expression

**Figure 1:**
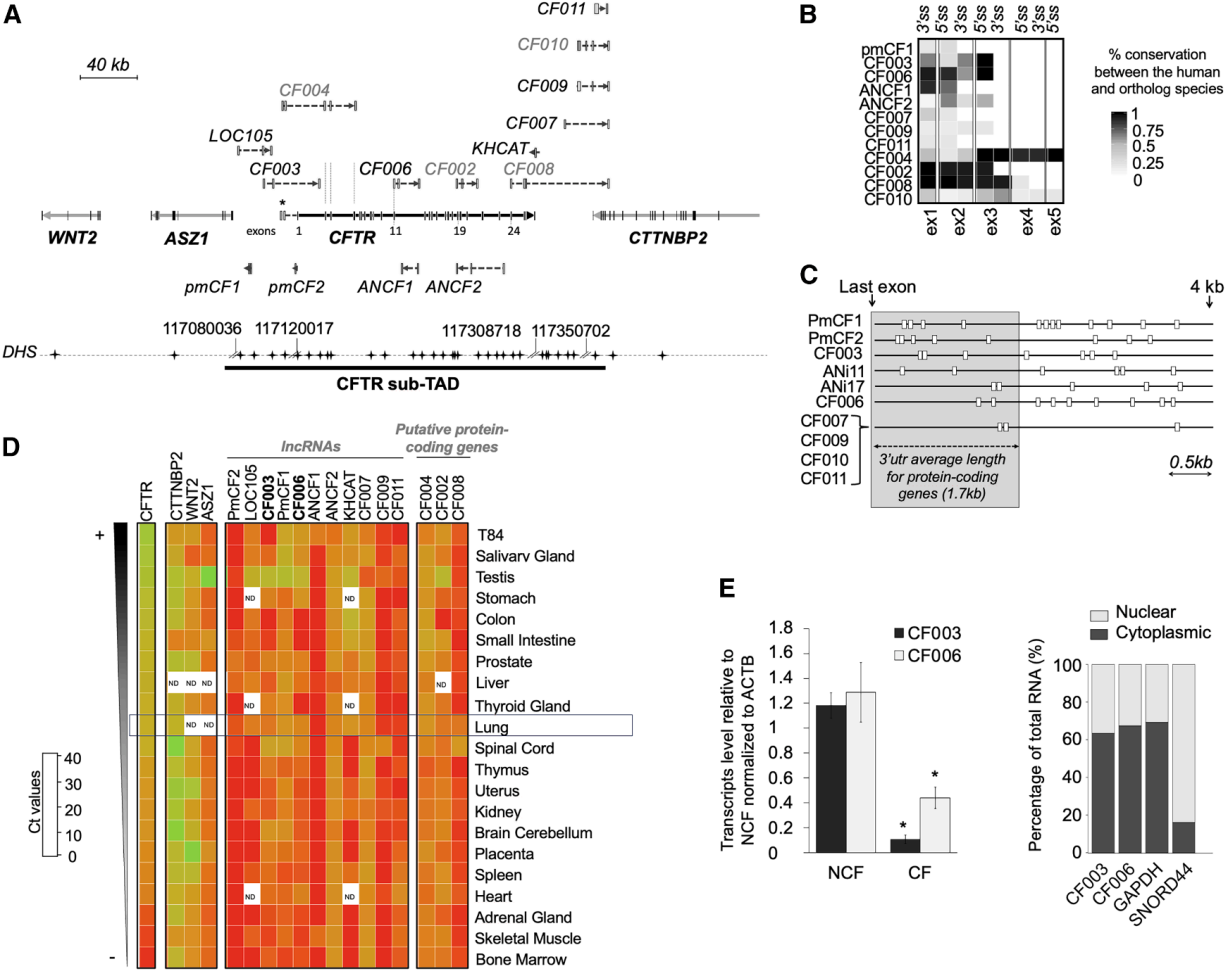
lncRNAs in the *CFTR* sub-TAD (A) Enlarged view of the *CFTR* sub-TAD including the neighboring genes (in bold). lncRNAs are in black and potential protein-coding genes are in light gray. TAD, topologically associated domain. Stars indicate DNase hypersensitive sites (DHSs) and correspond to the DHSs reported in the literature in lung cells. The reference sequence is the one reported in the Human Genome assembly GRCh37-hg19. (B) Heatmap showing splice-site (ss) conservation across orthologs. The sequences of each lncRNA were retrieved from the UCSC Genome Browser, and dinucleotides (canonical splice sites) were analyzed. Color code: white (non-conserved) to black (100% conserved, i.e., all species shared the same splice sites). (C) Results of the polyadenylation (poly(A)) signal prediction using RegRNA3. Poly(A) signals are indicated by white boxes. (D) Heatmap showing the expression levels of genes and lncRNAs identified in the *CFTR* sub-TAD assessed by quantitative PCR (Ct values without normalization). Color code is from red (no expression) to green (high expression). Tissues are ordered by *CFTR* expression level (from highest to lowest). ND, not determined. (E) Left panel: quantification of *CF003* and *CF006* in NCF and CF bronchial cells (*n* = 10 different NCF donors, *n* = 4 different CF donors) in air-liquid interface. Data were normalized to the *ACTB* transcript level. Right: *CF003* and *CF006* expression levels in the cytoplasmic and nuclear fractions of NCF bronchial cells (16HBE) obtained by qPCR. *GAPDH* and *SNORD44* were quantified as controls of the nuclear and cytosolic fractions, respectively. Significance was reported as follows: ∗*p* ≤ 0.05.

To identify new factors implicated in *CFTR* regulation, we interrogated different databases that collect known lncRNAs and the literature.^37^^,^^39^ We found eleven non-coding transcripts that overlapped the *CFTR* sub-TAD (Figure 1A; Table 1), including *BGas*, a previously reported lncRNA (named *CFTR-AS1* or *ANCF1*), involved in controlling *CFTR* expression by modulating the local DNA structure at its binding site.^37^ Specifically, we identified newly reported sense overlapping transcripts (*LOC105*, *CF003*, and *CF006*), natural antisense transcripts (*ANCF1* and *ANCF2*), promoter-associated RNA (*pmCF2*), and intergenic transcripts (*pmCF1*, *KHCAT*, *CF007*, *CF009*, and *CF011*). The *KHCAT*, *CF007*, *CF009*, and *CF011* lncRNAs were located at the 3′ extremity of the *CFTR* locus, and all shared the last exon of the *CTTNBP2* gene. As lncRNA sequence features could give clues on their functional properties, we explored their phylogenetic conservation. Compared with potential protein-coding genes in the same sub-TAD (*CF004*, *CF002*, and *CF008*), the canonical splice sites of these lncRNAs were weakly conserved across 17 ortholog species except for *CF003*, *CF006*, and *ANCF1* (Figure 1B). Furthermore, these *CFTR* lncRNAs harbored potential polyadenylation signals in their 3′ UTR, suggesting a possible export to the cytoplasm (Figure 1C). Next, we evaluated the expression of all these non-coding RNAs by qPCR in a broad range of human tissues using specific primers (Table 3) designed to span different exons, when possible. We also determined the expression levels of *CFTR* (including alternative and potential protein-coding forms) and neighboring genes (*CTTNBP2*, *ASZ1*, and *WNT2*). *CFTR* isoforms were modestly expressed across all tested human tissues (Figure 1D). Conversely, most of the screened lncRNAs were weakly expressed across human tissues (Figure 1D). Some non-coding transcripts were not expressed (*PmCF1*, *CF009*, and *CF011)*, while others were specifically restricted to a single tissue, such as *BGas/ANCF1* in skeletal muscle*.* Similarly, *CF003* and *CF006* displayed a marked expression in testis and salivary gland but were not expressed in the digestive system, where *CFTR* is well expressed. TSS sequencing (TSS-seq) analyses showed the presence of TSSs in the lung, testis, and colon for *CF003* and only in the lung and testis for *CF006*. Several natural TBP (TATA binding protein) motifs were predicted using the JASPAR tool for CF006 and CF003 (Figure S1A). We also assessed *CF003* and *CF006* expression levels in reconstituted epithelium cultured from bronchial cells from NCF (non-CF) individuals and individuals with CF harboring the p.Phe508del mutation. We observed a strong decrease in expression of *CF003* and *CF006* in CF cells compared with NCF cells (Figure 1E, left). As *CF003* and *CF006* represented strong *CFTR* lncRNA candidates, we decided to investigate their subcellular localization by qPCR. We detected them in both cytoplasmic (60%–65%) and nuclear (35%–40%) fractions of 16HBE cells (Figure 1E, right). Altogether, these data led us to focus on the *CF003* and *CF006* lncRNAs.

**Table 1:** Positions and source of identification for mRNA and non-coding RNAs

Name	Statut	Position (strand)	Length	Initial source (AnnoLnc ID, Ensembl); reference(s)	Status in LNCipedia 5
CF-Ex-1a	isoform	117119202 (+)	var	NA; Koh et al.,^9^ Lukowski et al.,^40^ and Mouchel et al.^41^	N/A
CF-Ex1aL	isoform	117119483 (+)	var	NA; Koh et al.,^9^ Lukowski et al.,^40^ and Mouchel et al.^41^	N/A
CF-Ex1a	isoform	117119516 (+)	var	NA; Koh et al.,^9^ Lukowski et al.,^40^ and Mouchel et al.^41^	N/A
CF002	PCT undefined	117251671 (+)	682	T27770, ENST00000468795.1	absent
CF004	PCT undefined	117119358 (+)	575	T27769, ENST00000446805.1	absent
CF008	PCT undefined	117292897 (+)	559	T27771, ENST00000600166.1	absent
CF-ANi11	NA	117204730 (−)	221	T2131	CFTR-AS1 (AC000111.3)
CF-ANi17	NA	117244845 (−)	376	T2136	lnc-CTTNBP2-1:1
CF-LNC	IT	117329767 (+)	423	T27468	absent
CF003	GAT	117105838 (+)	222	T27766, ENST00000546407.1	absent
CF006	GAT	117199692 (+)	148	T27765, ENST00000472848.1	absent
CF009	GAT	117350071 (+)	896	T27774, ENST00000608965.1	absent
CF010	GAT	117350186 (+)	519	T27775, ENST00000610149.1	absent
CF011	GAT	117353807 (+)	657	T27776	absent
AI80	PAT	117080036 (−)	421	T27768	absent
AN-pm	PAT	117119379 (−)	713	T27767	absent

Isoform, isoform including a surnumerous exon; N/A, not appropriate; PCT, protein-coding transcripts; NA, natural antisense; IT, intergenic transcript; GAT, gene-associated transcript; PAT, promoter-associated transcript.

### The CF003 and CF006 lncRNAs promote CFTR transcriptional activity

**Figure 2:**
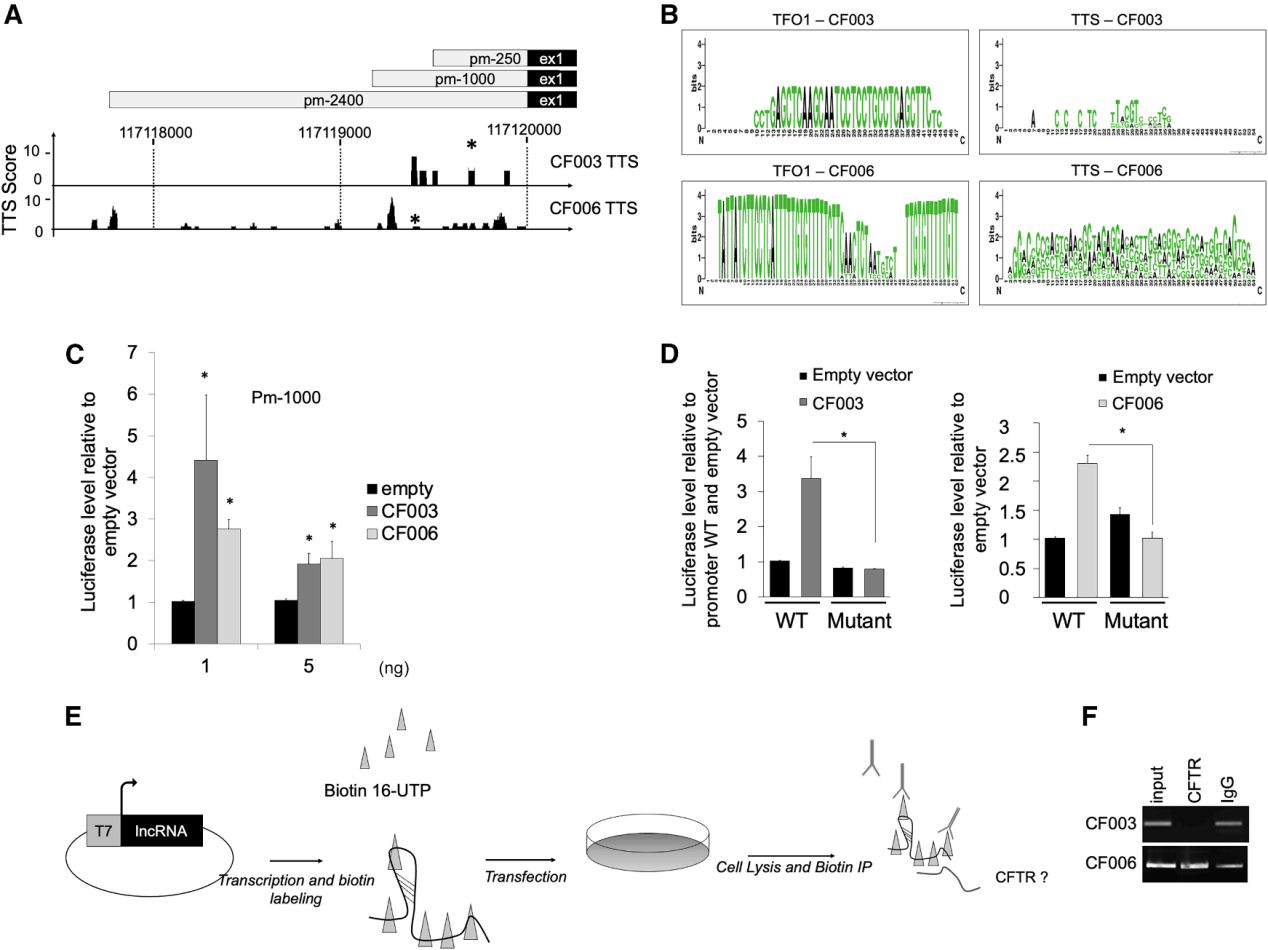
*CF003* and *CF006* positively regulate the *CFTR* promoter (A) *CF003* and *CF006* binding sites on the 2.3 kb (pm-2300), 1 kb (pm-1000), and 250 bp (pm-250) *CFTR* promoter regions predicted with LongTarget. The figure shows the predicted triplex target sites (TTSs). The peak height corresponds to the score strength of the TTS. (B) Logo sequences of triplex-forming oligonucleotide (TFO) and TTS sequences for *CF003* and *CF006* on the *CFTR* promoter. (C) Luciferase activity in BEAS-2b cells transfected with a reporter construct in which the luciferase gene is under the control of the 1 kb *CFTR* promoter or empty pGL3b vector and with *CF003* or *CF006* vector at 1 or 5 ng. Data were normalized to the empty vector. Significance was reported as follows: ∗*p* ≤ 0.05. (D) Luciferase activity in BEAS-2b cells transfected with a reporter construct in which the luciferase gene is under the control of the 1 kb CFTR promoter (wild type or harboring mutations in the strongest TTS motifs for *CF003* and *CF006*) or empty pGL3b vector and with 5 ng of *CF003* (left), *CF006* (right), or the empty vector. Data were normalized to the empty vector. Significance was reported as follows: ∗*p* ≤ 0.05. (E) Binding affinity of *CF003* and *CF006* for the *CFTR* promoter. lncRNAs were biotin labeled and co-transfected in BEAS-2b cells with the 1 kb *CFTR* promoter. (F) Binding affinity of *CF003* and *CF006* for the *CFTR* promoter. Chromatin immunoprecipitation was followed by amplification of the enriched DNA with primer sets against the *CFTR* promoter. Control: non-specific IgG.

lncRNAs can contribute to gene regulation by direct binding to the gene promoter and/or by affecting the binding of TFs (i.e., activators and/or repressors) or by acting on the chromatin architecture. lncRNAs mainly operate locally, near their sites of synthesis, on the host gene and neighboring genes, owing to their low expression. As *CF003* and *CF006* were modestly expressed and were detected in the nucleus, we decided to investigate their potential impact on *CFTR* promoter regulation, without ruling out the possibility that these lncRNAs could have functions in the cytoplasm. We predicted lncRNA-DNA binding sites for both *CF003* and *CF006* in the *CFTR* promoter using the LongTarget tool (with different *CFTR* promoter lengths) (Figure 2A). We identified several triplex target sites (TTSs) on the *CFTR p*romoter, particularly in the first 250 bp and up to 1,000 bp. Alignment of these motifs confirmed that the TTSs of *CF003* and *CF006* did not share similar sequences, but the triplex-forming oligonucleotides (TFOs) were quite similar (Figure 2B). To confirm these predictions, we assessed the impact of *CF003* and *CF006* overexpression on *CFTR* promoter activity using a luciferase reporter assay under the control of the 1 kb *CFTR* promoter. After overexpression of *CF003* or *CF006* at two concentrations (1 and 5 ng), luciferase activity increased in BEAS-2b cells in a dose-dependent manner (Figure 2C). We did not observe any effect of *CF003* or *CF006* overexpression when cells were transfected with the 2.4 kb or the 250 bp *CFTR* promoter (data not shown). These data suggested the presence of regulatory sequences for these lncRNAs in the 1 kb *CFTR* promoter. We also investigated the role of *CFTR*-lncRNAs on *CFTR* neighboring genes (CTTNBP2, ASZ, and WNT2), and we showed that overexpression of *CF003* and *CF006* increased *WNT2* expression in 16HBE cells (Figure S1B). Then, we mutated the strongest TTS motifs for *CF003* and *CF006* in the 1 kb *CFTR* promoter. Overexpression of *CF003* or *CF006* increased luciferase activity in cells transfected with the wild-type (WT) but not with the mutated 1 kb *CFTR* promoter (Figure 2D). This suggested the involvement of these TTS motifs in *CF003* and *CF006* recruitment to the *CFTR* promoter. Therefore, to determine whether *CF003* and *CF006* bound directly to the *CFTR* promoter, we co-transfected biotin-labeled *CF003* and *CF006* and the 1 kb *CFTR* promoter in BEAS-2B cells, and after immunoprecipitation, we amplified the enriched DNA using different primer sets (Figure 2E). The *CFTR* promoter was precipitated with *CF006* but not with *CF003* (Figure 2F). This indicates that only the *CF006* lncRNA binds directly to the *CFTR* promoter. Therefore, we focused on the *CF006* lncRNA.

### CF006 affects the binding of TFs on the CFTR promoter

**Figure 3:**
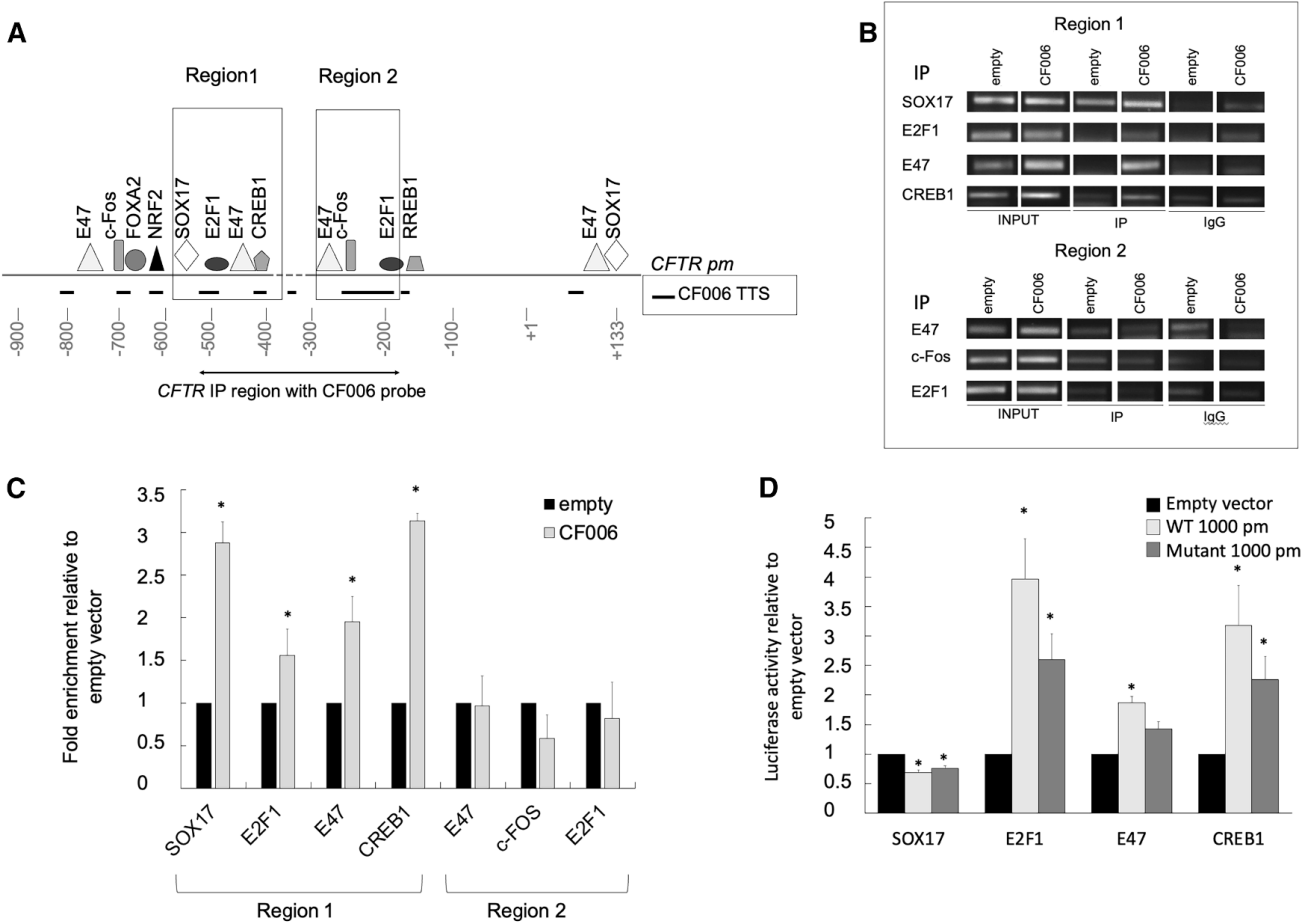
The *CF006* lncRNA is involved in *CFTR* gene regulation at the promoter level (A) Prediction of the binding sites of transcription factors (TFs) by JASPAR and their overlap with the TTS of *CF006* (black lines) in two regions of the 1 kb *CFTR* promoter. (B) Chromatin immunoprecipitation (ChIP) experiments in BEAS-2b cells transfected with empty vector (control, 250 ng) or *CF006* (250 ng) to test the interaction between *CF006* and the TFs identified in (A). Protein extracts were immunoprecipitated (IPed) with antibodies against the TFs indicated on the left or non-specific IgG (negative control) in regions 1 (top) and 2 (bottom). Input corresponds to the total lysate used as a control for PCR amplification of total DNA. (C) Quantification of the binding affinity of TFs for *CF006* (ChIP experiments described in B). DNA from immunoprecipitates and input DNA (5% of total chromatin) were analyzed by qPCR using primers that cover region 1 or 2 of the *CFTR* promoter. Data are shown as the fold enrichment of DNA after immunoprecipitation with the antibody against the indicated TF relative to the empty vector, set to 1. Significance was reported as follows: ∗*p* ≤ 0.05. (D) Effect of TFs on the *CFTR* promoter activity. Luciferase activity in BEAS-2b cells transfected with a reporter construct in which the luciferase gene is under the control of the 1 kb CFTR promoter (wild type or harboring a mutation in the strongest TTS motif for *CF006*) or empty pGL3b vector and with the indicated TFs. Data were normalized to the empty vector. Significance was reported as follows: ∗*p* ≤ 0.05.

*CF006* interaction with sequences on the *CFTR* promoter could affect the binding of local TFs on the *CFTR* promoter. To determine whether *CF006* was implicated in *CFTR* regulation in a coordinated way with TFs, we first used the JASPAR tool to predict the binding sites of several TFs on the 1 kb *CFTR* promoter and to determine whether their binding motifs overlapped with the TTS identified for *CF006* (Figure 3A). Then, to confirm their binding, we used chromatin immunoprecipitation (ChIP) experiments in bronchial cells to compare the binding of these TFs to the *CFTR* promoter in the presence or absence of the *CF006* lncRNA. In the presence of *CF006*, *SOX17*, *E2F1*, *E47*, and *CREB1* enrichment increased by 1.5–3 times compared with the empty vector (control), exclusively in region 1 (Figures 3B and 3C). These findings were obtained after PCR amplification of the DNA region captured with specific antibodies (Figure 3B) and after qPCR (Figure 3C). However, in our experimental conditions, E47, E2F1, and c-FOS (TFs predicted to bind to region 2 of the *CFTR* promoter) did not bind to region 2 (Figures 3B and 3C). Altogether, these data suggest that *CF006* binds to the *CFTR* promoter and affects the local binding of some TFs.

We also investigated the effect of TFs binding to region 1 on the 1 kb *CFTR* promoter activity (Figure 3D). Luciferase activity was increased by 2- to 3.5-fold upon overexpression of E2F1, E47, and CREB1, compared with the empty vector. Conversely, SOX17 overexpression decreased the luciferase activity, suggesting a repressive role on the *CFTR* promoter. Moreover, these TFs increased luciferase activity when cells were transfected with the TTS-mutant *CFTR* promoter, except E47 and SOX17 (Figure 3D). These data suggest that some TFs and *CF006* could be in competition for the same binding sites on the *CFTR* promoter.

### CF006 affects CFTR gene expression

**Figure 4:**
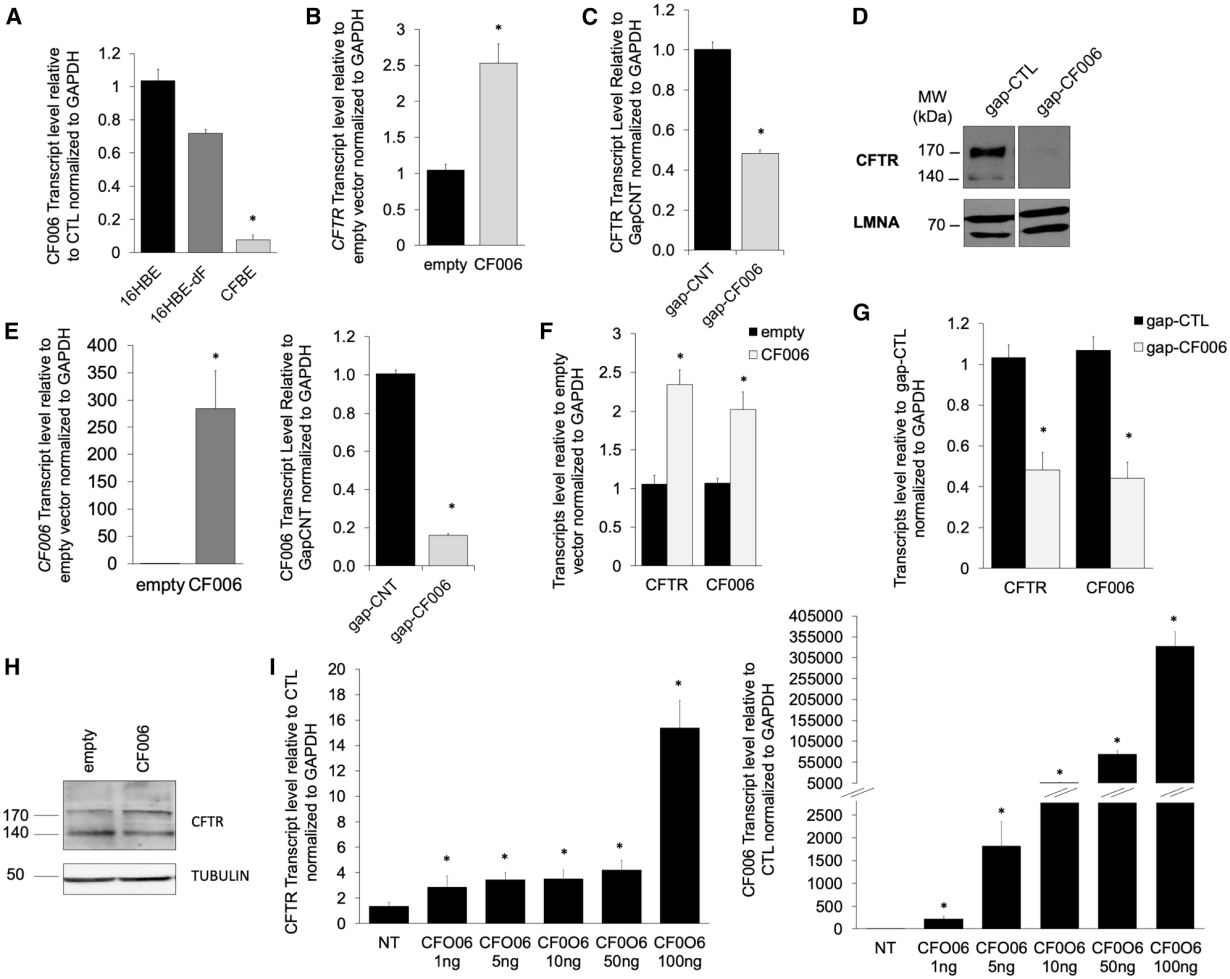
*CF006* promotes *CFTR* gene expression in CF cells (A) Quantification of endogenous *CF006* expression in 16HBE (NCF) and in 16HBE-dF and CFBE (CF models) cells. Data were normalized to the *GAPDH* transcript level. Significance was reported as follows: ∗*p* ≤ 0.05. (B) Quantification of *CFTR* mRNA level in 16HBE cells after *CF006* overexpression relative to empty vector. Significance was reported as follows: ∗*p* ≤ 0.05. (C) Quantification of *CFTR* mRNA level after *CF006* silencing using specific gapmeRs relative to control (gapCTL) in 16HBE cells. Data were normalized to the *GAPDH* transcript level. Significance was reported as follows: ∗*p* ≤ 0.05. (D) Western blot showing CFTR protein level in 16HBE cells after *CF006* silencing using specific gapmeRs (gap-CF006) compared with control (gapCTL). The endogenous LMNA protein level was used as the loading control. (E) Quantification of *CF006* in 16HBE cells after *CF006* transfection (25 ng) compared with empty vector (left) or *CF006* silencing (gap-CF006) compared with control (gapCTL) (right). Data were normalized to the *GAPDH* transcript level. Significance was reported as follows: ∗*p* ≤ 0.05. (F) Quantification of *CFTR* mRNA and *CF006* levels after *CF006* overexpression in comparison with empty vector in 16HBE-dF cells. Data were normalized to the *GAPDH* transcript level. Significance was reported as follows: ∗*p* ≤ 0.05. (G) Quantification of *CFTR* mRNA and *CF006* levels in 16HBE-dF cells after *CF006* silencing (gap-CF006) compared with control (gapCTL). Data were normalized to the *GAPDH* transcript level. Significance was reported as follows: ∗*p* ≤ 0.05. (H) Western blot showing CFTR protein level after *CF006* overexpression or not (empty vector) in 16HBE-dF cells. Endogenous tubulin protein level was used as the loading control. (I) Quantification of *CFTR* mRNA and *CF006* levels in primary CF cells (hAEC-CF) after exogenous *CF006* transfection compared to non-treated cells. Data were normalized to the *GAPDH* transcript level. Significance was reported as follows: ∗*p* ≤ 0.05.

Our results using the luciferase reporter system suggested that *CF006* acts as an activator upon its recruitment to the *CFTR* promoter region. Therefore, we assessed whether in CF cells, *CF006* recruitment affected endogenous *CFTR* expression. First, we measured *CF006* expression levels in 16HBE cells and in two CF cell models, CFBE cells (derived from a patient with CF) and 16HBE-dF. *CF006* was decreased in both 16HBE-dF and CFBE cells compared with 16HBE cells (NCF control) (Figure 4A). We then assessed the *CF006* effect on *CFTR* expression in 16HBE and 16HBE-dF cells, as they share the same background. *CF006* transfection induced an increase in *CFTR* transcript levels by ∼2.5-fold in 16HBE cells (Figure 4B). To corroborate these data, we designed LNA gapmeRs to inhibit *CF006* expression. In 16HBE cells, the gapmeRs targeting *CF006* (gapCF006) significantly decreased *CFTR* mRNA expression by 50% (Figure 4C) and also CFTR protein levels (Figure 4D). We obtained the same results in T84 cells that display high CFTR expression (Figure S1C). In 16HBE-dF cells (CF model), *CF006* overexpression increased the *CFTR* transcript level by 2- to 2.5-fold (Figure 4F, left) as well as the CFTR protein level (Figure 4H). Conversely, *CF006* silencing with gapCF006 decreased *CFTR* expression by 2- to 2.2-fold (Figure 4G, left). Next, we assessed the *CF006* effect on *CFTR* mRNA expression in CF primary cells (CF human airway epithelial cells [hAEC-CFs]) cultured in monolayers (Figure 4I). We confirmed *CF006* overexpression and silencing in 16HBE cells (Figure 4E) and in both 16HBE-dF cells and hAEC-CFs (Figures 4F, 4G, and 4I, right). Subcellular localization of CF006 was detected in primary hAECF-CFs, confirming nuclear (62%) expression (Figure S2A). Altogether, these data confirmed that *CF006* acts as an activator of *CFTR* gene expression in both NCF and CF cells.

### miR-145 influences CFTR gene expression by binding to CF006

**Figure 5:**
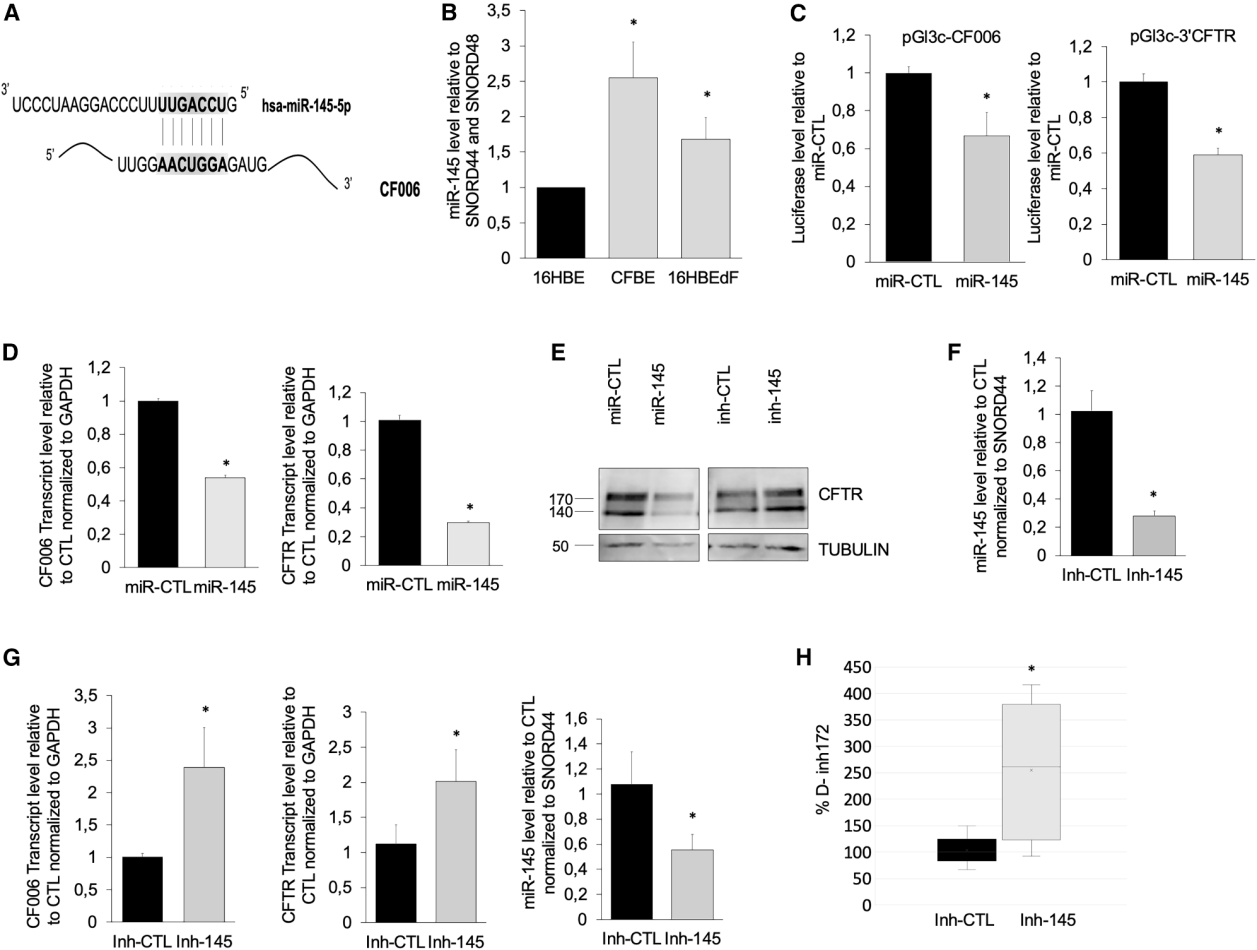
*CF006* expression is regulated by miR-145 (A) miR-145 binding motif in the *CF006* lncRNA sequence detected using the TargetScan software. (B) Quantification of endogenous miR-145 expression in 16HBE (NCF) and 16HBE-dF and CFBE (CF) cells. Data were normalized to the *SNORD44* and *SNORD48* transcript levels. Significance was reported as follows: ∗*p* ≤ 0.05. (C) Luciferase activity in BEAS-2b cells transfected with a reporter construct in which the luciferase gene is under the control of *CF006* (left) or the 3′ UTR of *CFTR* and with miR-145 or miR-CTL. Significance was reported as follows: ∗*p* ≤ 0.05. (D) Quantification of endogenous *CF006* (left) and *CFTR* (right) mRNA levels after miR-145 overexpression compared with miR-CTL in 16HBE cells. Significance was reported as follows: ∗*p* ≤ 0.05. (E) Western blot showing CFTR protein level in 16HBE cells after miR-145 overexpression or miR-145 silencing (inh-145) compared with miR-CTL or inh-CTL, respectively. Endogenous tubulin protein level was used as the loading control. (F) Quantification of miR-145 levels in 16HBE cells after miR-145 silencing (inh-145) compared with inh-CTL. Significance was reported as follows: ∗*p* ≤ 0.05. (G) Quantification of *CF006* (left), *CFTR* mRNA (middle), and miR-145 (right) levels in ALI-CF cells (epithelium cultured from bronchial cells from patients with CF) after miR-145 silencing (inh-145) compared with inh-CTL (*n* = 4, refers to two different donors and two different filters, Student’s *t* test). Significance was reported as follows: ∗*p* ≤ 0.05. (H) Graph showing the mean of the maximal inhibition (percentage) of CFTR in CF cells after miR-145 silencing (inh-145) compared with inh-CTL (*n* = 4, refers to two different donors and two different filters, Student’s *t* test). Significance was reported as follows: ∗*p* ≤ 0.05.

It was previously reported that several lncRNAs are deregulated by miRNAs.^42^ Using TargetScan, we predicted the presence of miRNA binding sites for hsa-miR-145-5p (miR-145 hereafter) on *CF006* (Figure 5A). We and others have already demonstrated that miR-145 expression is upregulated in CF cell cultures and that it controls CFTR expression by binding to the *CFTR* 3′ UTR.^7^ We hypothesized that in CF cells, miR-145 could have a dual action on CFTR expression by binding to the *CFTR* 3′ UTR and to *CF006*. To this aim, first, we quantified *miR-145* expression levels in 16HBE cells (NCF) and in CFBE and 16HBEdF cells (CF models). Its expression was increased by 1.5- to 2.5-fold in 16HBEdF and CFBE cells compared with 16HBE cells (Figure 5B). Next, we determined the impact of miR-145 overexpression on *CF006* and *CFTR* expression in BEAS-2B cells using plasmids in which the cDNA luciferase is under the control of the *CF006* sequence and of the *CFTR* 3′ UTR sequence, respectively. miR-145 overexpression induced a decrease in luciferase activity compared with control in both conditions (Figure 5C). We next confirmed in 16HBE cells that miR-145 overexpression decreased *CF006* and *CFTR* transcript levels (Figure 5D). In agreement, miRNA-145 overexpression decreased CFTR protein levels, and miR-145 silencing (inh-145) increased CFTR protein levels in 16HBE cells (Figure 5E). We confirmed miR-145 silencing after inh-145 transfection in 16HBE cells (Figure 5F). In air-liquid interface CF (CF-ALI) cells, miR-145 silencing (inh-145) (Figure 5G, right) induced an increase in *CF006* and *CFTR* mRNA levels (Figure 5G, left and middle). Similar results were obtained in CF primary hAEC-CFs, with an increase in both CF006 and *CFTR* mRNA expression levels in CF primary cells (hAEC-CFs) (Figure S2B). Lastly, we assessed the CFTR channel activity by using Ussing chamber-based measurements. Overexpression of *CF006*, after miR-145 silencing, significantly increased CFTR activity (Figure 5H). Altogether, these data confirmed the dual role of miR-145 on *CFTR* expression by directly acting on the 3′ UTR of *CFTR* and on the lncRNA *CF006*.

## Discussion

We investigated the presence of lncRNAs within the CFTR sub-TAD (i.e., CFTR locus and its adjacent regulatory regions spanning approximately −50 kb to +50 kb), a chromatin domain insulated by boundaries and characterized by cell-type-specific enhancer-promoter looping.^25^ Most of the screened lncRNAs were poorly expressed in human tissues, similarly to what was reported for the vast majority of lncRNAs.^30^^,^^43^^,^^44^^,^^45^ Both *CF003* and *CF006* lncRNAs in the *CFTR* locus displayed tissue-specific expression and induced *CFTR* expression in CFTR-expressing cells and acted as negative regulators in cells that express low CFTR levels. *BGas* (*ANCF1*), a previously reported lncRNA that controls CFTR through chromatin remodeling, also displayed very low expression in human tissues.^38^ As *CF003* and *CF006* were mainly enriched in the nuclear compartment, we focused on their transcriptional role, but we are not ruling out the possibility that they could have important roles in the cytoplasm by affecting the post-transcriptional regulation of *CFTR* through interaction with RNA-binding proteins or miRNA sponging.

In this study, we also investigated the role of *CFTR*-lncRNAs on *CFTR*-neighboring genes. Our data suggest a function of both *CF003* and *CF006* on *WNT2* expression in the lung. *CF006* seemed to positively act on *WNT2* expression in bronchial cells, a key regulator of lung development. Moreover, the TFs NRF2, FOXA2, RREB1, and SOX17 in pulmonary cells are negative regulators, whereas CREB1, AP-1, E2F1, and USF are positive regulators of *CFTR* expression.^7^^,^^16^ We showed that binding of *CF006* on the *CFTR* promoter altered the recruitment of TFs involved in lung development, such as FOXA2, which we previously highlighted as involved in *CFTR* expression switch-off from the fetal to the adult stage.^7^ The strong expression of *CF006* in fetal compared with adult lung tissue suggests a role at this stage. More studies are needed to determine whether these lncRNAs contribute to the decrease in CFTR expression from the fetal to the adult stage and to assess their importance in lung development through *regulation of CFTR* and *WNT2*. Altogether, these data underscore a more complex cooperative regulation involving TFs, miRNAs, and lncRNAs to modulate *CFTR* expression in bronchial cells and perhaps during lung development. Aberrant lncRNA expression has been reported in various diseases, including CF. Prior studies have shown deregulation of multiple lncRNAs in airway cells,^46^ suggesting their potential as disease markers. Here, we demonstrate that CF003 and CF006 are downregulated in CF primary airway cells and bronchial cell lines. Importantly, CF006 overexpression significantly increased CFTR levels in CF bronchial cells, positioning it as a promising therapeutic target to enhance CFTR expression and function.

We also show that miR-145, previously known to bind the CFTR 3′ UTR and repress its expression,^7^^,^^19^ exerts a dual effect by also targeting CF006. miR-145 overexpression reduced both CF006 and CFTR levels, while its inhibition led to increased expression of both transcripts and enhanced CFTR channel activity in 3D CF epithelium. These results underscore the interplay between miRNAs and lncRNAs in regulating CFTR and suggest that targeting miR-145 could indirectly restore CF006 levels and improve CFTR function. A major challenge remains the efficient delivery of lncRNA-based therapies. Previous work has shown that Tat-mediated delivery or exosome encapsulation may be viable strategies for lncRNA or miRNA inhibitor administration in CF contexts.^47^

In conclusion, our findings emphasize the importance of deciphering CFTR enhancers and silencers to better understand its sophisticated regulatory landscape. Identifying non-coding elements such as CF006 opens new avenues for therapeutic intervention, potentially complementing existing CFTR modulators or benefiting patients with mutations not currently addressed by approved treatments. Moreover, these insights may extend to other lung disorders where CFTR expression is reduced.

## Materials and methods

### *In silico* analyses

lncRNAs in the *CFTR* locus and adjacent regulatory regions (−50 kb, +50 kb) were extracted from the UCSC Genome Browser (hg19), AnnoLnc,^48^ Ensembl Database (GRCh37), LNCipedia5,^39^ and NONcode v.3.0 databases.^49^ *CFTR* spliced transcripts were also studied as control. Presence in LNCipedia5 was assessed, and reference numbers were added when available (Table 1). All CFTR isoforms and lncRNAs in the *CFTR* locus are listed in Table 1. Polyadenylation signals for each transcript were predicted with the RegRNA 3.0 web server (https://awi.cuhk.edu.cn/∼RegRNA). A phylogenetic study was carried out to determine the sequence conservation at splicing sites across mammals using the Multiz Align tool from the UCSC Genome Browser (human, rhesus, baboon, mouse lemur, mouse, rat, guinea pig, squirrel, rabbit, dolphin, cow, horse, cat, dog, elephant, chicken, and sheep). TBP binding sites were predicted using the JASPAR web-based tool (https://jaspar.elixir.no). TSSs were retrieved using cap analysis of gene expression (CAGE) data from airways cells and TSS-seq data from lung, colon, and testis tissue samples, deposited in the UCSC Genome Browser.

### Primer design

Primers for lncRNA detection were designed using Primer3 (http://primer3.ut.ee/) and their specificity was validated with Primer-BLAST (http://www.ncbi.nlm.nih.gov/tools/primer-blast/) and Nucleotide Collection. lncRNA primers were selected in two different exons for multi-exon transcripts. Amplifications were carried out with MasterMix (Promega, France), and PCR products were controlled on agarose gel and sequenced on an ABI-3130XL Genetic Analyzer using the Big Dye Terminator v.1.1 Cycle Sequencing Kit (Applied Biosystems). To ensure the correct comparison between tissues and cell lines, primers for the housekeeping genes *GAPDH* and *ACTB* were also used.

### Cell culture

Nasal cells were obtained, after informed consent, from patients with CF harboring the p.Phe508del mutation and individuals without CF (NCF). Cells were cultured in the ALI as previously described.^7^ Bronchial cells from NCF and CF individuals grown in the ALI (CF-ALI) system were purchased from Epithelix (Epithelix, Switzerland). Both cell types were cultured in PneumaCultExPlus medium (Life Technologies, St Aubin, France) supplemented with 5 mM L-glutamine, 1% antibiotics, and 10% fetal bovine serum (FBS; Eurobio, France). The 16HBE14o- (16HBE; human bronchial cell line), CFBE41o- (CFBE; derived from a human CF bronchial tissue sample harboring the homozygous p.Phe508del mutation), and 16HBEge-p.Phe508del (16HBE-dF, gene-edited 16HBE cells harboring the p.Phe508del and referenced as CFF-16HBEge CFTR F508del(M470)) cell lines were cultured in MEM (Thermo Scientific, France) supplemented with 10% FBS, 1% penicillin/streptomycin, and 1% L-glutamine. BEAS-2b (human bronchial cells) and T84 (derived from a colon carcinoma lung metastasis) cells were grown in high-glucose DMEM medium (Life Technologies) supplemented with 5% FBS, 1% antibiotics, 1% L-glutamine, and 1% Ultroser. All cells were maintained at 37°C and 5% CO_2_. CF primary cells (hAEC-CFs) were derived from a homozygous p.Phe508del-CFTR patient (EP57AB-CFAB060901, Epithelix, female, 21 years old), and hAEC-CFs were cultured in complete PneumaCultExPlus medium with 1% antibiotics. All cell lines are described in Table 2.

**Table 2:** Cell lines and primary cells used

Name	Status	Type	Provided by
BEA-2B	non-CF	bronchial	ATCC
T84	non-CF	epithelial colic cell	ATCC
Bronchial MucilAir	non-CF/CF	primary bronchial differentiated	Epithelix
16HBE	non-CF	bronchial cells	Gruenert’s lab
16HBEge-F508del	p.Phe508del homozygous	bronchial cells	CFF Foundation
CFBE41o-	p.Phe508del homozygous	bronchial cells	Gruenert’s lab
hAEC-CF	p.Phe508del homozygous	primary bronchial cells	Epithelix

### Gene reporter constructs, expression vectors, and site-directed mutagenesis

The *CF003* and *CF006* sequences were amplified from cDNA of 16HBE cells and subcloned in the pTracer-CMV2 expression vector (kind gift by Pascale Fanen, Créteil, France) using the KpnI and XbaI restriction enzymes. The 2.3 kb *CFTR* promoter sequence was previously described,^4^ and the 1 kb *CFTR* promoter sequence was obtained from Panomics (Redwood, USA). Mutations in the top DNA-binding motifs of *CF003* and *CF006* were introduced in the 1 kb *CFTR* promoter with the QuikChange II Site-Directed Mutagenesis Kit (Agilent, Courtaboeuf, France). The 1 kb promoter sequence was then subcloned into the pm-pcDNA3.1-CFTR-3′ UTR plasmid to generate the pm1kb-pcDNA3.1-CFTR-3′ UTR construct. All constructs were verified by direct sequencing. The expression vector for SOX17 in the pcDNA3.1 plasmid was previously described.^7^ The expression plasmids for E2F1 and E47 were from Addgene, and CREB1 and c-FOS expression plasmids were purchased from Origene (Herford, Germany).

### Transient transfection of the expression vectors and LNA gapmeRs

For both transcript and protein analyses, cell lines were seeded in 6-well plates (250,000 to 350,000 cells per well). 25 ng of each lncRNA-coding plasmid was transfected with JetPei transfection reagent (PolyPlus, Illkirch, France) in 16HBE and 16HBE-dF cells. LNA gapmeRs (Exiqon, Vedbaek, Denmark) that specifically target each lncRNA were designed and transiently transfected using RNAiMAX (Life Technologies) at a final concentration of 50 nM. Cells were harvested 24 h post-transfection for transcript analysis and 48 h post-transfection for protein analysis. Cells were seeded (250,000 cells per well) in 6-well plates. At 70%–80% confluency, exogenous lncRNA (transcribed from lncRNA-coding plasmid with the T7 RiboMAX Express Large Scale RNA Production System [Promega] and purified with TRIzol reagent [LifeTech]) was transiently transfected using RNAiMAX at the indicated concentrations in hAEC-CFs for 24 h.

### RNA isolation and quantitative PCR analysis

Total RNA was extracted using TRIzol reagent (LifeTech). Cytoplasmic and nuclear RNA fractions were isolated using the SurePrep Nuclear or Cytoplasmic RNA Purification Kit (Fisher Scientific, Illkirch, France). Total RNA from 20 human tissues was obtained from ClonTech (Ozyme, Saint-Quentin-en-Yvelines, France). Then, total RNA from adult tissues, cell lines, and primary cells (500 ng–1 μg) was reverse transcribed with the M-MLV Reverse Transcriptase Kit (Invitrogen, Villebon sur Yvette, France), and cDNA abundance was measured with the SYBR Green master mix (Roche, Basel, Switzerland) on a Light Cycler 480 (Roche) according to the manufacturer’s instructions. The expression of lncRNAs and mRNAs was determined by normalization with the reference genes *ACTB* and *GAPDH* and the 2^(−ΔΔCt)^ method.^50^ Primers for PCR-based quantification are listed in Table 3.

**Table 3:** Primers for the amplification of isoforms and non-coding RNAs associated with CF

Name	Forward	Reverse	Length (bp)
CF-Ex-1a	CCCTTGGAGTTCACTCACCT	ATTCCAGGCGCTGTCTGTAT	var.
CF-Ex1a	TGCCAACTGGACCTAAAGAGA	ATTCCAGGCGCTGTCTGTAT	var.
CF-Ex1aL	TCAAAATGCACCTTGCAAAC	ATTCCAGGCGCTGTCTGTAT	var.
CF004	GTTCTTTTCTCCGACACGCA	GCGTTCCTCCTTGTTATCCG	567
CF003	GTGCCCCATCAACTGGACTT	TTCCAGGCGCTGTCTGTATC	202
CF002	GGACGGCAGCCTTACTTTGA	GTTCAAATCTCACCCTCTGGC	526
CF-ANi11	AACAGATCAACTTTGGCAGTCA	AAGGCCTTCTTCCAGGAATAGT	165
CF-ANi17	GAGACCCAACTCCAAAAGCA	GGGATTTGCCCACATTTCTA	235
CF-LNC	AAGCAAGCAGAGGGTTTTGA	TGGGTTAGATGCCCAGTTTC	223
CF006	TGGCTAGTGGTTTGGGAAAA	GTCCTGCCTGCTCCTATGAC	140
CF008	TTGGATCCCTATGAACAGTGG	GGTAAGAGTTTCCTGGGTTAGTTG	431
CF009	GAGCATTGATAAATTGAACACAA	CAATGCCTCCATTGAAGAGG	169
CF011	TGGCTAGTGGTTTGGGAAAA	CAATGCCTCCATTGAAGAGG	194
AI805947	TGCTTTGCGTAATTACCGGC	TGCCCCTCAGAGAGTTGAAG	267
AN-pm	TGAAGATGAGGAGCAAGATGA	CAGAAGCAAGATGCAATGGA	393

### Western blotting

Whole proteins were extracted with 1× Laemmli buffer and separated on 7% SDS-polyacrylamide gels. After transfer onto PVDF membranes and a blocking step with 5% skimmed milk in 0.1% Tween 20/PBS (T-PBS), membranes were incubated (4°C, overnight) with the anti-CFTR Ab432 (1:500) or Ab450 (1:500) antibodies, obtained from the Cystic Fibrosis Foundation, or with the anti-LMNA (1:1,000, Millipore, #05-714) or TUBULIN-beta (1:3,000, ProteinTech, #66240) antibody. Then, membranes were washed with T-PBS and incubated with horseradish peroxidase (HRP)-conjugated mouse IgG binding protein (1:10,000, sc-516102, SantaCruz) at room temperature. After washes with T-PBS, proteins were revealed using the Immobilon Western Chemiluminescent HRP substrate (Millipore).

### Luciferase assays

BEAS-2b and 16HBE cells were seeded in 96-well plates (10,000 and 20,000 cells per well, respectively). *CFTR-1kb*-promoter or *CFTR*-2.3kb-promoter subcloned in the luciferase-encoding pGL3-Basic reporter vector were co-transfected at 72 ng with the *CF003* or *CF006* expression plasmid (1 and 5 ng). For the mutant experiments, the 1 kb *CFTR* promoter harboring the mutated motif for the top binding site of *CF003* or *CF006* was used. For the TF experiments, the 1 kbp *CFTR* promoter in the pGL3-Basic reporter vector was co-transfected with the expression vectors encoding SOX17, E47, E2F1, CREB1, or c-FOS at different concentrations. For all assays, the control vector pRL-SV40 encoding Renilla luciferase was co-transfected at 10 ng per well. 48 h post-transfection, cells were lysed, and luciferase activity was measured using the Dual-Luciferase Reporter Assay System (Promega) on a Luminoskan Ascent luminometer (ThermoLabsystem, France). Results correspond to the ratio of luciferase to Renilla activity normalized to empty control plasmids.

### Prediction of lncRNA, TF, and miRNA binding sites in the CFTR promoter

DNA-binding sites for *CF003* and *CF006* were predicted using the 1 and 2.3 kb *CFTR* promoter sequences and the LongTarget tool (http://lncrna.smu.edu.cn/). TTSs were obtained as well as the corresponding TFOs. TTS position and score were plotted as custom tracks in the UCSC Genome Browser. The TTSs with the highest score, specific to the 1 kbp *CFTR* promoter, were mutated. TF binding sites were predicted using the 1 kbp *CFTR* promoter sequence and the JASPAR tool. The 80% score threshold was applied, and only TFs with binding sites within 100 bp of the potential binding region of the lncRNAs *CF003* and *CF006* were reported. The TargetScan website (https://www.targetscan.org) was used to predict miRNA binding sites in the CF006 sequence.

### Biotin-labeled CF006 and CF003

Biotin-labeled *CF006* and *CF003* were generated using the T7 polymerase and the Biotin RNA Labeling Mix (Roche) according to the manufacturer’s instructions. Templates for T7 transcription were prepared by PCR and the pTCF-CF006 or pTCF-CF003 plasmid. Control biotin-labeled RNA was obtained by digesting the pcDNA3.1-USF1 plasmid with HincII to produce a 315 bp fragment. All probes were stored as ethanol precipitates at −80°C.

### ChIP assays

ChIP experiments were carried out using the Millipore kit (Kit 17-295, Merck, Saint Quentin en Yvelines, France) and as previously described.^8^ For TFs, 1.10^6^ BEAS-2b cells were seeded, and after 24 h, they were transiently transfected with 250 ng of control or *CF003*- or *CF006*-encoding plasmid. For biotin immunoprecipitation, BEAS-2b cells were co-transfected with the pm1kb-pcDNA3.1-CFTR-3′ UTR plasmid (1.5 μg) and biotin-labeled lncRNAs (500 ng). For all assays, 24 h after transfection, cells were incubated with 5 mM dimethyl 3,3′-dithiobispropionimidate for 30 min to stabilize protein-protein interactions and then cross-linked with 1% formaldehyde at room temperature for 5 min. Cells were scraped in PBS supplemented with a proteinase inhibitor cocktail (Sigma, P8340) and pelleted. Chromatin was sheared by sonication (Bioruptor) and incubated at 4°C with antibodies (from SantaCruz) against SOX17 (sc-130295), CREB1 (sc-377154X), E47 (sc-390376X), E2F1 (sc-251X), and rabbit IgG (sc-2027; negative control). Immunoprecipitated DNA and input were analyzed by semi-quantitative PCR using primers that encompassed the binding regions of the corresponding factors. Experiments were repeated twice and normalized to the negative control and input. PCR products were loaded in 1.5% agarose gels.

### Ussing chamber-based measurements

Bronchial epithelial cells were cultured in filters (filtration area, 0.33 cm^2^), and the hsa-miR-145-5p inhibitor (inh-145) or negative control (inh-CTL) was added using peptide nanoparticles, as previously described.^47^ First, to inhibit the apical epithelial Na+ channel, the sodium (Na+)-channel blocker amiloride (100 μM) was added. To activate transepithelial cAMP-dependent currents including Cl transport through CFTR channels, the cAMP agonists forskolin (FSK; 10 μM) and IBMX (100 μM) were added. Next, the potentiators of CFTR activity VX-770 (1 μM) and genistein (10 μM) were added, followed by the addition of the CFTR gating inhibitor (CFTR_inh-172_, 10 μM) to specifically inhibit CFTR. Lastly, to challenge the purinergic calcium-dependent chloride secretion, ATP (100 μM) was added. Data represent the maximal CFTR inhibition (in percentage) after incubation with CFTR_inh-172_ (*n* = 4).

### Statistical analysis

Luciferase and RT-qPCR assays were carried out at least three times, and each experiment was analyzed in triplicate. *p* values were determined using the non-parametric Wilcoxon (unpaired, Mann-Whitney) test or unpaired two-sample Welch’s *t* tests for luciferase or RT-qPCR assays, respectively (∗*p* ≤ 0.05). All the data were normally distributed (Shapiro-Wilk test), and SEM data are shown.

## Data and code availability

All data supporting the findings of this study are available from the corresponding author upon request.

## Acknowledgments

French ethical research committee approval for nasal cells collection from patients, N°ID-RCB 2011-A01520-41. This work was supported by grants from the French association Vaincre La Mucoviscidose (RF20180502291 and RF20190502506) and FEDER-10.13039/100010137FSE-IEJ 2014/2020 (M.T.-C.).

## Author contributions

K.D. performed most of the experiments with J.V., who carried out mainly the luciferase, IP, and RNA analyses. K.D. carried out the Ussing chamber-based measurements. A.B. and P.B. contributed substantially to revising the manuscript. M.T.-C. initiated and managed the entire project.

## Declaration of interests

The authors declare no conflicts of interest.

## Supplemental information

Document S1. Figures S1 and S2

Document S2. Article plus supplemental information
