# Supplementary material for: Long non-coding RNAs in the CFTR locus: New transcriptional regulators of CFTR gene expression
Source: Mol Ther Nucleic Acids. 2026 Jul 27;37(3):103039. doi: 10.1016/j.omtn.2026.103039 (PMC13476513; doi:10.1016/j.omtn.2026.103039)
Supplement: Supplementary file 1 [file mmc1.pdf]

OMTN, Volume *37*

Supplemental information

Long non-coding RNAs in the CFTR locus: New transcriptional regulators of CFTR gene expression

Jessica Varilh, Karine Deletang, Arnaud Bourdin, Prisca Boisguerin, and Magali Taulan-Cadars

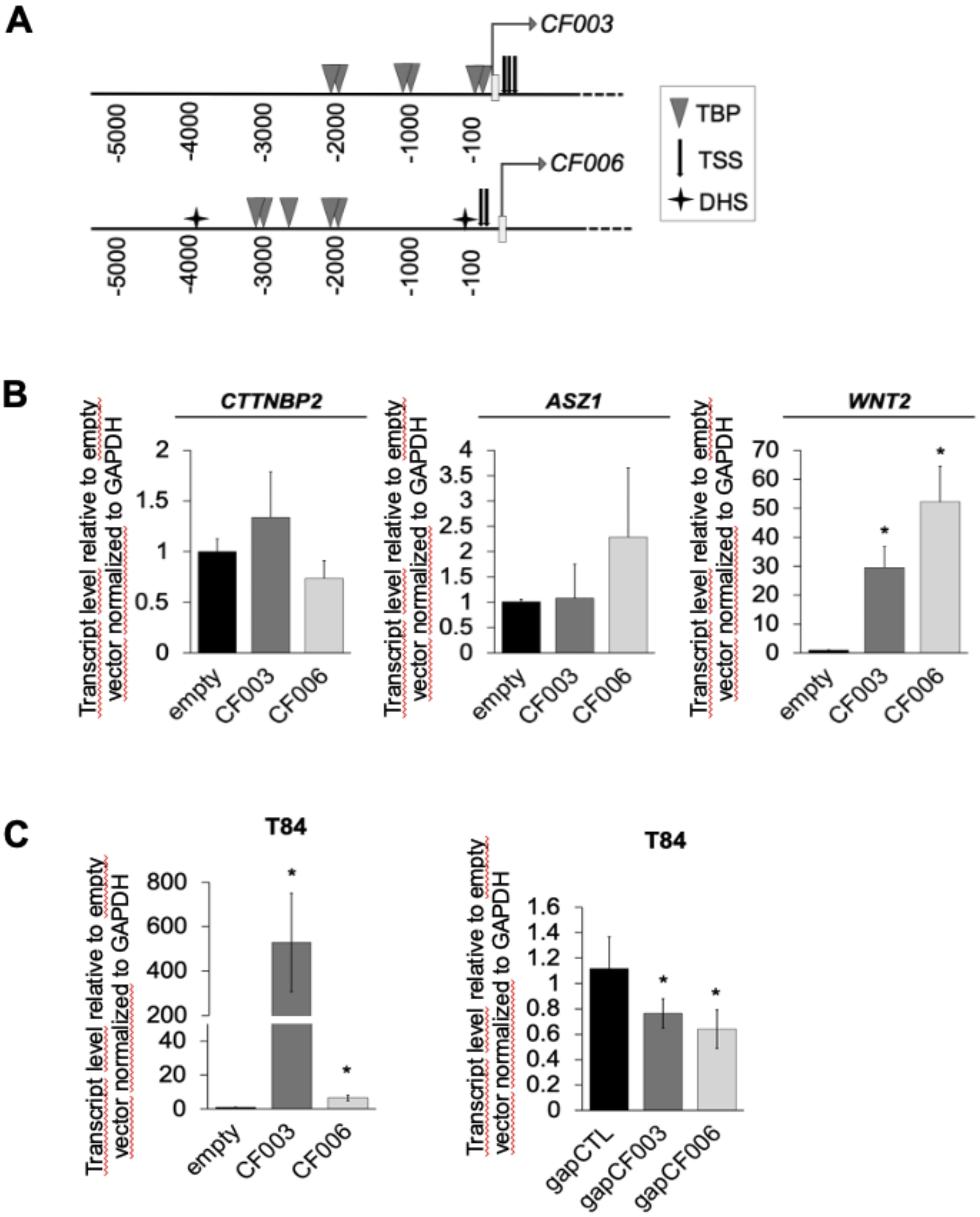

Figure S1:

A. Prediction of Transcriptional Start Sites (TSS) using CAGE data in lung, colon and testis, of TATA-binding protein (TBP) sites, using JASPAR prediction, and of DNase Hypersensitive Sites (DHS), using literature data, for CF003 (top panel) and CF006 (bottom panel).

B. Quantification of CTTNBP2 (left panel), ASZ1 (middle panel), WNT2 (right panel) in 16HBE cells after overexpression of CF003 and CF006 in comparison with empty vector). Data were normalized to GAPDH transcript level. Significances were reported as followed *≤0.05.

C. Quantification of CF003 and CF006 expression in T84 cells either after CF003 or CF006 overexpression (left panel) or after CF003 (gapCF003) or CF006 (gapCF006) silencing (right panel) in compared with control (gapCTL). Data were normalized to GAPDH transcript level. Significances were reported as followed *≤0.05

A

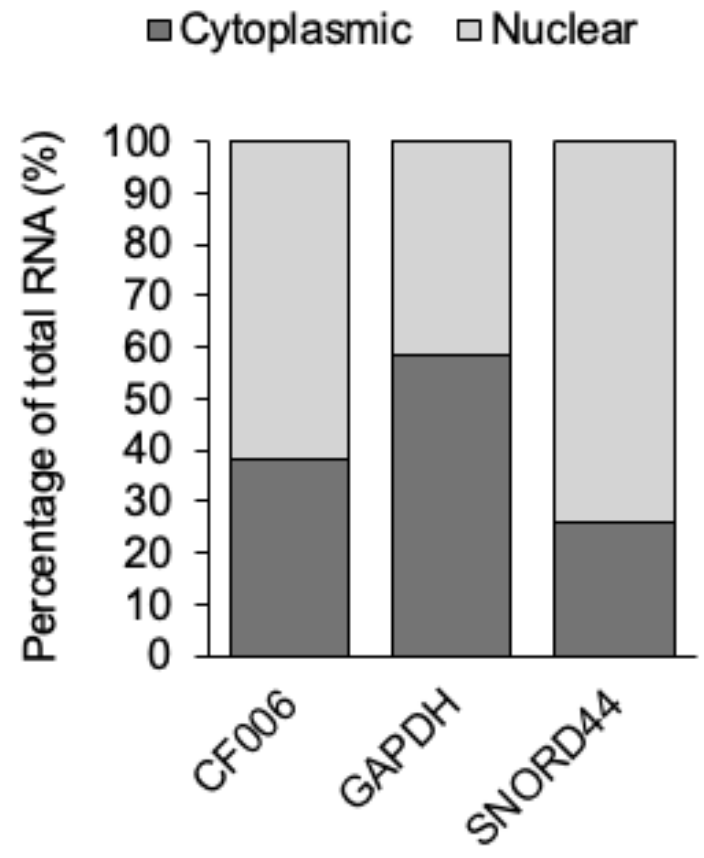

B

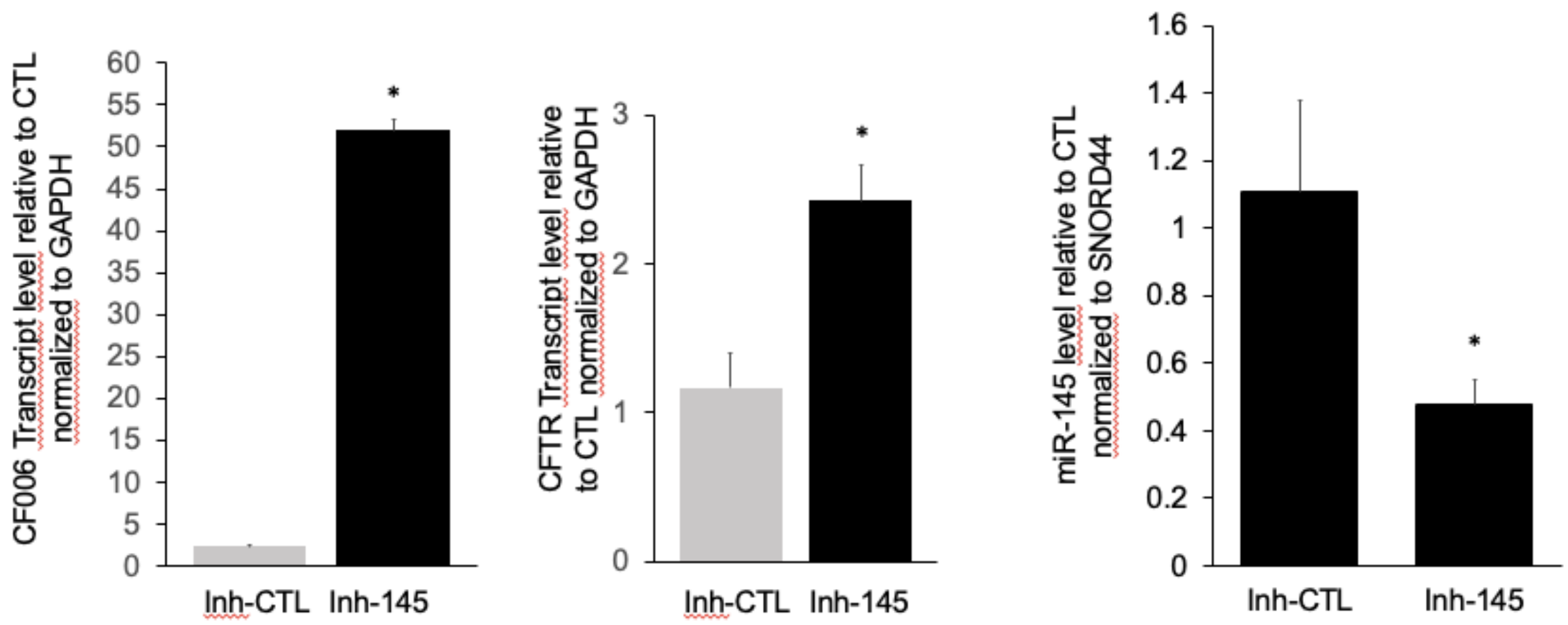

Figure S2:

A. CF006 expression levels in the cytoplasmic and nuclear fractions of primary CF cells (hAEC-CF) obtained by qPCR. GAPDH and SNORD44 were quantified as controls of the nuclear and cytosolic fractions, respectively.

B. Quantification of CF006 (left), CFTR mRNA (middle) and miR-145 (right) levels in primary CF cells (hAEC-CF) after miR-145 silencing (Inh- 145) compared with inh-CTL (n = 4, Student's t test). Significances were reported as followed *≤0.05.
